# Implementation of IoT-Based Air Quality Monitoring System for Investigating Particulate Matter (PM_10_) in Subway Tunnels

**DOI:** 10.3390/ijerph17155429

**Published:** 2020-07-28

**Authors:** Jun Ho Jo, ByungWan Jo, Jung Hoon Kim, Ian Choi

**Affiliations:** 1 Department of Civil and Environmental Engineering, Hanyang University, Seoul 04763, Korea; kpxjuno08@hanyang.ac.kr (J.H.J.); kimj32@hanyang.ac.kr (J.H.K.); 2 Yongsan International School of Seoul, Seoul 04347, Korea; ianchoi1013@gmail.com

**Keywords:** air quality monitoring, particulate matter, subway tunnels, Internet of Things, cloud computing

## Abstract

Air quality monitoring for subway tunnels in South Korea is a topic of great interest because more than 8 million passengers per day use the subway, which has a concentration of particulate matter (PM_10_) greater than that of above ground. In this paper, an Internet of Things (IoT)-based air quality monitoring system, consisting of an air quality measurement device called Smart-Air, an IoT gateway, and a cloud computing web server, is presented to monitor the concentration of PM_10_ in subway tunnels. The goal of the system is to efficiently monitor air quality at any time and from anywhere by combining IoT and cloud computing technologies. This system was successfully implemented in Incheon’s subway tunnels to investigate levels of PM_10_. The concentration of particulate matter was greatest between the morning and afternoon rush hours. In addition, the residence time of PM_10_ increased as the depth of the monitoring location increased. During the experimentation period, the South Korean government implemented an air quality management system. An analysis was performed to follow up after implementation and assess how the change improved conditions. Based on the experiments, the system was efficient and effective at monitoring particulate matter for improving air quality in subway tunnels.

## 1. Introduction

The subway is a major transportation system in South Korea used by approximately eight million commuters per day [1,2]. In order to secure a comfortable environment for passengers, it is important to investigate air quality in the system [3]. While the subway has provided environmentally sustainable transit by reducing traffic congestion on the road, it has become evident that subway systems have air pollution problems. Such problems may present potential health risks to commuters and working staff because the concentration level of particulate matter (PM_10_) is generally higher than that above ground, proven by published studies [4,5,6,7,8,9,10].

According to many researchers, most aerosol particles in subway tunnels are by-products of friction between the wheels of an operating subway car and the train rails. Unlike pollutants above ground, particulate matter in subway tunnels is generally composed of metals such as iron, copper, magnesium, zinc, silicon, barium, and manganese [7,11,12,13,14]. Additionally, air pollutants are carried by natural air circulation or commuters. The government has been diversifying its efforts to improve the air quality of subway systems by installing air purifiers, installing wind guard doors, and conducting national research projects [15,16,17,18]. Unfortunately, increased amounts of PM_10_ have been deteriorating lung function and causing respiratory difficulties [19,20,21,22,23]. It is clear that investigating the contamination level of air quality in subways, especially with PM_10_, is required to provide basic data for establishing an improvement plan.

In this study, an Internet of Things (IoT)-based air quality monitoring system was developed to monitor particulate matter and was implemented in a subway tunnel in Incheon, South Korea. The main purpose of this system was to monitor particulate matter in real-time and to analyze the condition of PM_10_. In this study, IoT technology was used to measure and transmit air quality data, while cloud computing technology was applied to analyze and store data. In addition, an instant alert system was developed to inform personnel to take immediate action when a change in air quality was detected.

## 2. IoT-Based Air Quality Monitoring System

**Figure 1:**
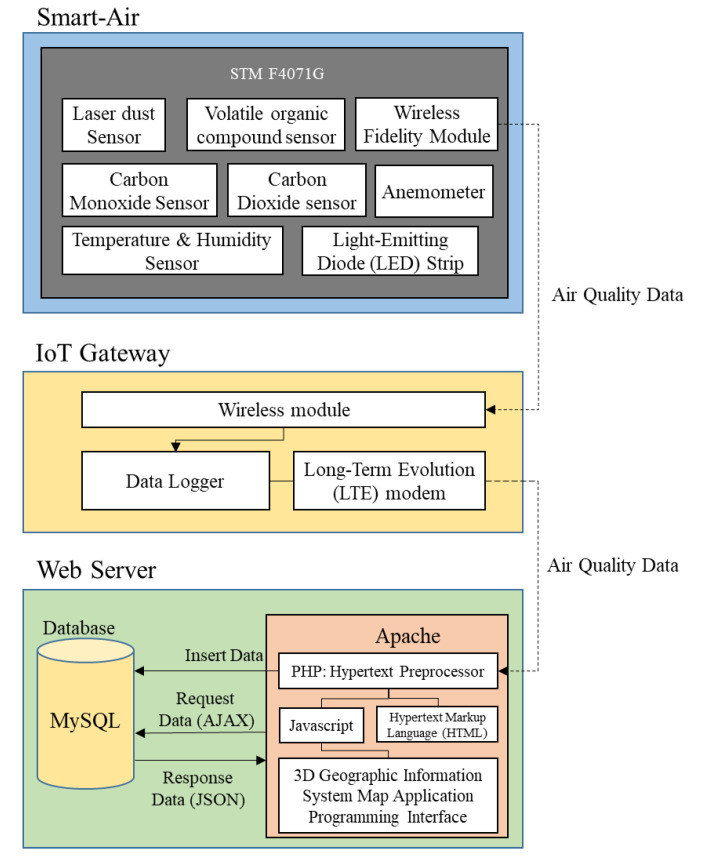
A configuration diagram of the Internet of Things based air quality monitoring system.

In order to monitor air quality accurately and efficiently in real-time, an IoT-based air quality monitoring system was developed for subway tunnels to monitor air quality by measuring the concentration levels of particulate matter. As shown in Figure 1, the system was primarily divided into three parts: A Smart-Air device, an IoT gateway, and a cloud computing-based web server. We used Smart-Air devices for the measurement of PM_10_ to monitor and determine air quality in subway tunnels. The gateway was used as a bridge, wirelessly connecting the Smart-Air device with the web server. The web server allowed us to analyze and determine air quality conditions. It has the ability to calculate the Air Quality Index (AQI) for particulate matter, to visualize the concentrations of each pollutant, and to store the data for further analysis [24].

### 2.1. Smart-Air

**Figure 2:**
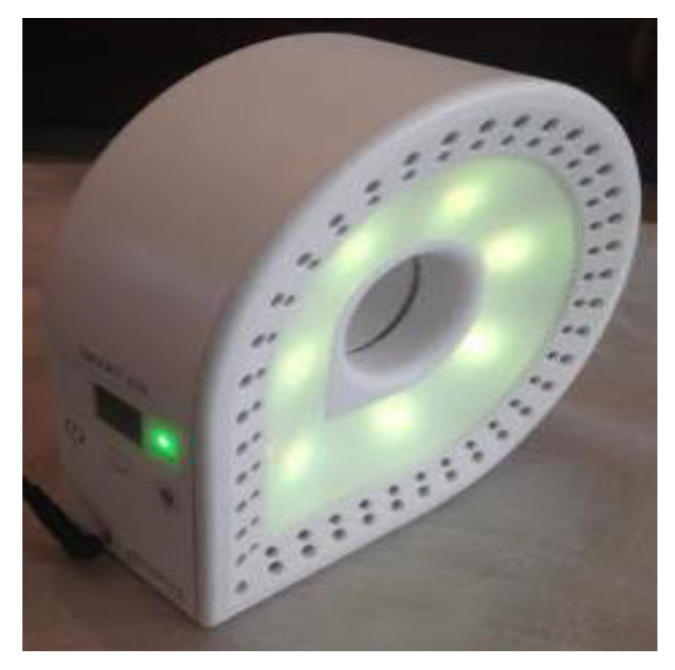
Smart-Air device.

The Smart-Air device is an air quality monitoring device from Smart-Is that is designed to monitor indoor air quality with accuracy and reliability by using an expandable interface as shown in Figure 2. Therefore, multiple sensors can be easily installed or edited according to monitoring requirements. Moreover, a Light-Emitting Diode (LED) strip mounted in the device displays different colors to indicate the condition of air quality in real-time. The color code for this display is defined by AQI.

Since the Smart-Air device has an expandable interface, it can measure any pollutants, including other particulate matter beyond PM_10_, through installation of appropriate sensors in the device. In addition, it is possible to install several devices to expand coverage when the monitoring area is too large for only one device. However, the device was designed to measure concentrations of PM_10_, and a laser dust sensor mounted in the device was used in the research. In the device, a laser dust sensor (PM 2007, from Wuhan Cubic Opto-electronics Co.) was installed to measure particle sizes from 0.3 to 10 μm. The sensor was successfully verified for reliability by following the prescribed procedure from the Ministry of Environment, Korea [24].

To test the reliability of the laser dust sensor mounted in the device, a combination of a chamber experiment and field test were performed based on concentration of particulate matter. The sensor data on the Smart-Air device were compared to a different certified particulate matter measurement device to test the reliability. The Korean Ministry of Environment recommended and requested that light-scattering particulate matter measurement device from GRIMM Aerosol Technik Ainring GmbH & Co. KG was used due to its ability to detect concentration of particulate matter by a light-scattering method, the most reliable method for detection. Three Smart-Air devices and a GRIMM 1109 device were placed in an acrylic chamber. Then, external air was injected at a rate of 1L/min and then 2.5L/min. Data were measured at 1, 30, and 60 min marks as seen in Table 1. The readings of the Smart-Air devices were compared to those of the GRIMM 1109 device to assess the reliability of the Smart-Air devices [24].

In each experiment, the three Smart-Air devices, labeled Smart-Air (a), Smart-Air (b), and Smart-Air(c), measured the concentration of particulate matter. The three devices showed constant and stable values. In addition, the devices were very similar to the GRIMM 1109 device. At 1L/min and 30 min, the Smart-Air devices and GRIMM 1109 device both measured 93 μg/m. At 2.5L/min and 30 min, the Smart-Air devices and GRIMM 1109 device measured a similar concentration of 97 μg/m. As shown through the experiments, the Smart-Air devices measured similar concentrations of fine dust to the GRIMM 1109 device. This indicates that the laser dust sensor on Smart-Air devices has a high reliability [24].

### 2.2. IoT Gateway

Since multiple Smart-Air devices were installed for monitoring, a wireless network was required to connect the devices with the web server. The IoT gateway gathers data from multiple devices and transmits the data to the web server for monitoring purposes. The gateway consists of a wireless module and a data logger to transmit and gather the sensing data. Two types of wireless modules are mounted in the gateway: a Wi-Fi module and a Long-Term Evolution (LTE) module. The data from each device are gathered via Wi-Fi and transferred in the form of Transmission Control Protocol/Internet Protocol (TCP/IP) packets to the web server via LTE. Then, the data are analyzed through the web server for visualization while being saved in a database.

### 2.3. Web Server

The web server is designed to visualize concentrations of each pollutant and to calculate AQI for particulate matter. The web server stores data in the database to support future analysis that may be required, such as for monthly or annual air quality monitoring analysis.

In this research, the cloud-computing-based web server from Amazon Web Services (AWS) was used to manage measurement of the air quality efficiently and effectively. AWS is a certified commercial cloud computing platform that offers open source libraries for various applications, such as an alarming service and a cloud storage service. Thus, using AWS reduces the time and cost needed to create customized versions of these applications. In addition, AWS provides the highest standard of security to protect data stored in the database.

For research, an Apache web server was designed with a web programming language called hypertext preprocessor, while MySQL was used as the database. Moreover, EC2 (elastic compute cloud) was used for programming interfaces to support configuration of the virtual machine instance and dynamic instantiation. For an extensible instance among EC2, a t2.medium server was used for the web server with the specifications shown in Table 2 [25,26].

## 3. Methods

**Figure 3:**
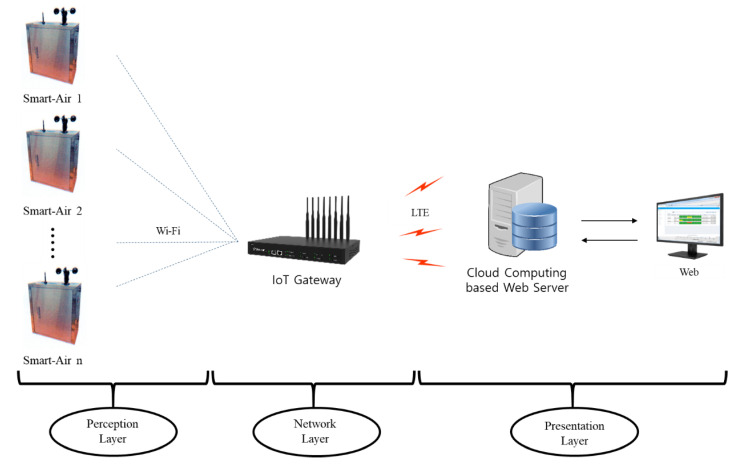
A system diagram of the IoT-based air quality monitoring system.

The monitoring method in this study was based on the IoT monitoring platform. The IoT platform is a network of physical devices that interconnects objects within systems, using networking technologies to make more intelligent interactions between each object [27]. By integrating IoT technology into a monitoring platform, sensors are directly connected with a monitoring system to provide real-time analysis of particulate matter in the tunnel. The IoT platform comprises three main components: a perception layer, a network, and a presentation layer, as shown in Figure 3 [28,29,30]. For the perception layer, multiple Smart-Air devices were used to detect air quality, with a focus on particulate matter. The measurements collected from the Smart-Air devices were transmitted to the gateway via Wi-Fi. The gateway was the network layer of the platform, and the Smart-Air devices were connected to the cloud computing web server via LTE. Then, the web server analyzed the received data to determine air quality and visualize the AQI for particulate matter. Using the cloud server, managers or related personnel could monitor particulate matter levels at any time, from anywhere.

This research focused on implementing an IoT-based air quality monitoring system in subway tunnels. An additional research goal was to analyze the contamination level and determine the state of the air quality regarding particulate matter in the tunnel. The experiment was performed between January 2017 and January 2019 in selected subway tunnels in Incheon, South Korea.

### 3.1. Monitoring Locations

Between 2012 and 2017, the South Korean government implemented a subway facility maintenance project to enhance air quality in subway stations [1]. The maintenance included installing air purifiers, replacing asbestos used in the tunnels, installing screen doors and wind guard doors to block the inflow of fresh air from outside, and improving the filter system. By 2018, the ventilation operating time was changed to 20 h per day, and stations began to stop the ventilation system temporarily (to block airflow from outside of the tunnel) when the concentration of particulate matter was 150 µg/m^3^ or more outside. Since Smart-Air devices were installed and collected data between Bupyeongsamgeori station and Dongsu station, we analyzed the change in the concentration level of particulate matter in the tunnel in the course of the maintenance project.

**Figure 4:**
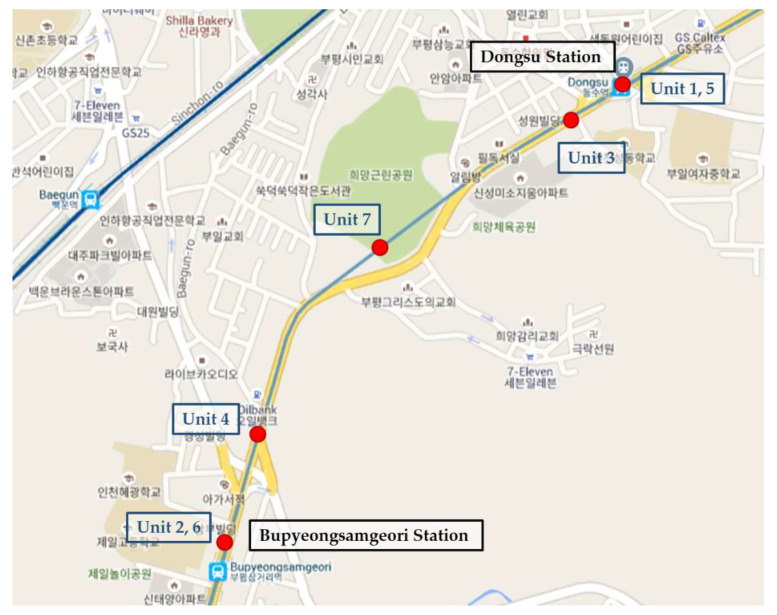
Monitoring locations in Incheon, Korea.

For this research, seven Smart-Air devices were installed from Bupyeongsamgeori station to Dongsu station as shown in Figure 4. Two devices (units 1 and 2) were installed at the center of each station platform to monitor the air quality of the platforms. Two other devices (units 3 and 4) were installed on the starting points of each tunnel to collect data as trains entered or left the stations. In addition, two devices (units 5 and 6) were installed near the central ventilation rooms of each station, and the last device (unit 7) was installed in the center of the tunnel to measure the change in values when train speeds were at their maximum.

### 3.2. Cloud-Computing-Based Web Server

**Figure 5:**
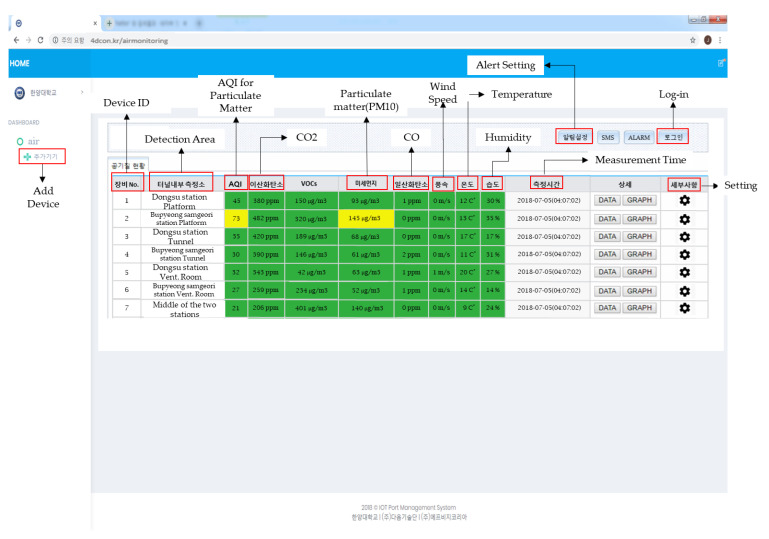
Cloud computing web server of the IoT-based air quality monitoring system.

In this research, the most essential role of the web server was to analyze the measurements received from Smart-Air devices. The web server (as shown in Figure 5) visualized the data and calculated AQI based on PM_10_. Then, the web server communicated with the sensors installed in the devices to display concentrations of particulate matter through the LED strips. In addition, the AQI and concentrations of PM_10_ were stored in the server database for detailed analysis.

The data were classified according to the ID and location of the devices in the web server. Graphs and data sheets from stored data could be extracted for detailed analysis. The data were displayed with color based on the AQI. The web server was designed to alert users by pop-up message when air quality was moderate or poor, so that users could take immediate action if needed.

### 3.3. Air Quality Index

The AQI for particulate matter was calculated using data from Smart-Air devices connected to the web server. The AQI is a value defined by the United States Environmental Protection Agency (EPA) to characterize the quality of air. Concentrations of PM_10_ were used to compute the AQI, which is described in Equation (1) [31]:

(1) $$I=\left({C\;-\;C}_{l}\right)\frac{I_{h}-I_{l}}{C_{h}-C_{l}}{+I}_{l}$$

where I = Air Quality Index, C = rounded concentration of pollutant (PM_10_), C_h_ = concentration breakpoint that is greater than or equal to C, C_l_ = concentration breakpoint that is less than or equal to C, I_h_ = AQI value corresponding to C_h_, and I_1_ = AQI value corresponding to C_l_.

Furthermore, each level of health concern is assigned a range of AQI and color code as shown in Table 3 [31,32]. In this research, the Smart-Air devices display colors to passengers who are in the monitored area. In addition, the web server was set up to alert managers by pop-up message on a smartphone when the level of health concern is below good, so that immediate action can be taken.

## 4. Results

The goal of this study was to implement an IoT-based air quality monitoring system in a subway tunnel in order to investigate the condition of particulate matter. The AQIs for particulate matter were successively analyzed and displayed on the web server with a 95% confidence level. The results showed that the AQI and concentration of particulate matter varied based on location and time, even in the same tunnel. In addition, data on the AQI for particulate matter (AQI data from 2017 and 2018) demonstrated that air quality conditions improved after implementing the national maintenance project.

### 4.1. Analysis of AQI

**Figure 6:**
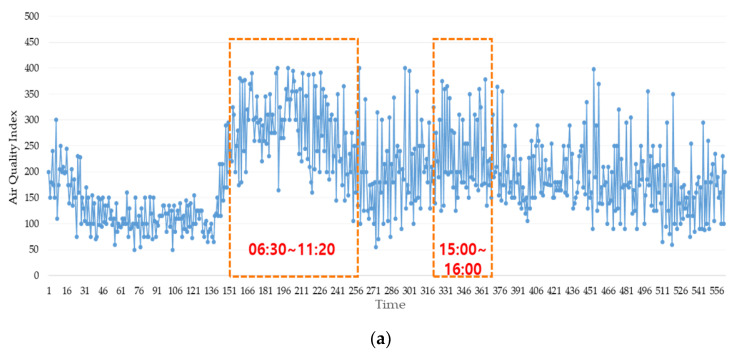

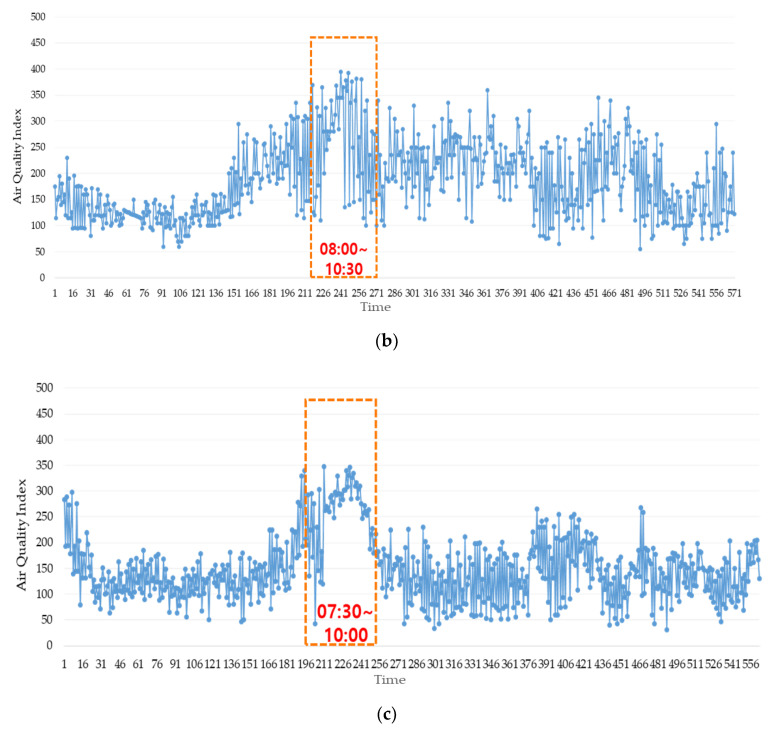
AQI for particulate matter (PM_10_) measured in (**a**) Dongsu main ventilation room, (**b**) Bupyeongsamgeori station tunnel, and (**c**) Dongsu station tunnel.

The air quality in terms of particulate matter was analyzed using the AQI from each of seven monitoring locations. The highest (worst) AQI measured was 405, in Dongsu’s main ventilation room, as shown in Figure 6a. In the same room, the highest concentrations of particulate matter were observed for 5 h in the morning (from 06:30 to 11:20) and an hour in the afternoon (from 15:00 to 16:00), which were the longest residence times at the monitoring locations. The second highest AQI was detected in Dongsu tunnel, close to the main ventilation room. In this case, particulate matter had a residence time of 2.5 h (08:00 to 10:30), as shown in Figure 6b. Analysis of AQI revealed that the residence times of particulate matter were longer in locations deeper underground. The depth of Dongsu’s main ventilation room is 30 m, the deepest of all measurement locations. In addition, Bupyeongsamgeori is located 25 m underground, and it showed a residence time of 2.5 h, as depicted in Figure 6c. Since high concentrations of particulate matter seem to stay longer in subway tunnel locations that are deep underground, it is necessary to change air quality management systems to create better airflow and decrease concentrations and residence times of particulate matter.

### 4.2. Analysis of Particulate Matter Concentration

**Figure 7:**
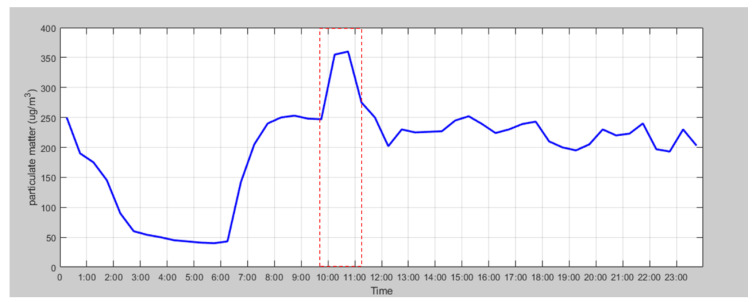
Daily concentration of PM10 in Bupyeongsamgeori station tunnel.

To investigate air quality for particulate matter during subway operation times, data measured from Bupyeongsamgeori station tunnel were analyzed and are shown in Figure 7. The operating hours of the subway are from 05:37 to 00:37. The concentration of PM_10_ decreased from 01:00 and reached the lowest value between 02:30 and 03:00, when trains were not operating. The concentration level remained unchanged after operating hours and gradually decreased after an hour. According to the Ministry of Environment, Korea, the permissible hourly PM_10_ concentration is 80 µg/m^3^. The ministry recommends refraining from long-term activities in the area when the concentration is over the limit and avoiding any activities when the concentration is higher than 150 µg/m^3^ for PM_10_ [33]. Although the concentration was measured in the tunnel where only trains operate, the concentration was only measured below the limit when the trains were not in service, which shows that train operation has an impact on the concentration of PM_10_. Likewise, the increase in the concentration level did not occur right after trains started operating. The concentration of PM_10_ in the tunnel slowly increased from 07:00 to 09:00, i.e., during rush hours. Furthermore, the maximum concentration was measured between morning and afternoon rush hours after 10:00. After that peak, it seems that particulate matter followed the air flow generated by operation of trains.

### 4.3. Annual Comparison of Particulate Matter Concentration

**Figure 8:**
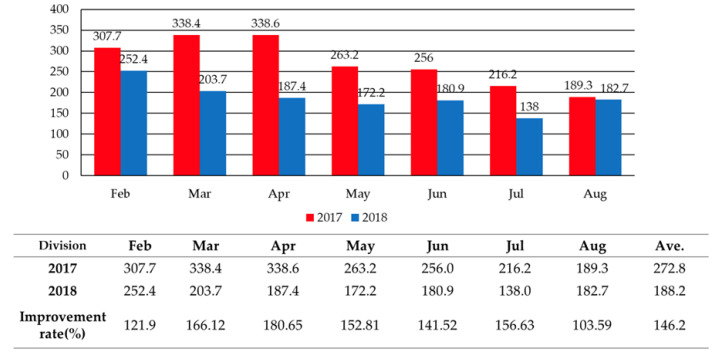
Monthly PM_10_ concentrations in Bupyeongsamgeori station tunnel.

From 1 February 2017 until 31 August 2018, the measurement of real-time particulate matter PM_10_ concentration was conducted in front of Bupyungsamgori station platform to measure the change in concentration in the tunnel. As mentioned, the analysis was performed to see whether changes in the management system affected air quality, by comparing the concentration level before and after the changes were implemented. The results of the analysis are depicted in Figure 8. The 2018 particulate matter concentration showed an improvement of 146.2% when compared to 2017, proving that the new management system was effective. It is considered that particulate matter concentration was reduced due to airflow in the tunnel (created by changes in the management system, including operating the ventilation system for 20 h per day and utilizing wind guard doors).

## 5. Conclusions

In this paper, the implementation of an IoT-based air quality monitoring system to improve the monitoring of particulate matter (PM_10_) levels was presented. For the experiments, the system was implemented in subway tunnels in Incheon, South Korea. The results demonstrated good performance of the system and convenience of monitoring air quality with a focus on particulate matter. Several conclusions were drawn, including the following: (1) The IoT-based air quality monitoring system efficiently monitored air quality in the tunnel. (2) The air quality in the subway tunnel could be monitored consistently using the IoT and cloud computing technology. (3) It was easy to expand the monitoring area by extending the system.

The following observations were also made: (1) The particulate matter in deeper locations had longer residence times. (2) The maximum concentration of particulate matter occurred between the morning and afternoon rush hours. (3) The air quality improved significantly when the operation time of the ventilation system was changed to 20 h per day, and the system was temporarily stopped when the concentration of particulate matter from outside of the tunnel was 150 µg/m^3^ or more.

More tests are necessary to exclude or correlate additional factors, such as the presence of other pollutants, the detailed impact of tunnel ventilation, and varying environments in the tunnel. In addition, an automatic air quality management application (a convergence of artificial intelligence and big data) can be added. These technologies would be able to automatically predict and improve air quality in subway systems.

## Figures and Tables

**Table 1:** Measurements from the reliability test of the laser dust sensor.

	Burn Flow: 1L/min			Burn Flow: 2.5L/min		
	1st	2nd	3rd	1st	2nd	3rd
GRIMM 1109 (μg/m^3^)	93			97		
Smart-Air (a) (μg/m^3^)	92	93	92	95	96	97
Smart-Air (b) (μg/m^3^)	92	93	92	96	97	96
Smart-Air (c) (μg/m^3^)	92	93	91	96	97	96

**Table 2:** Specifications of the t2.medium for AWS.

Properties	T2.Medium
Processor	3.3 GHz Intel Scalable Processor
vCPU	2
Storage	Elastic Block Storage-Only
Memory (GiB)	4
CPU Credits/hour	24
Network Performance	Low to Moderate

**Table 3:** Classifications of Air Quality Index.

AQI Values	Levels of Health Concern	Color Codes
(When the AQI is in This Range:)	(…Air Quality Conditions Are:)	(…As Symbolized by This Color:)
0 to 50	Good	Green
51 to 100	Moderate	Yellow
101 to 150	Unhealthy for Sensitive Groups	Orange
151 to 200	Unhealthy	Red
201 to 300	Very Unhealthy	Purple
301 to 500	Hazardous	Maroon
